# Novel antimicrobial activities of a peptide derived from a Japanese soybean fermented food, Natto, against *Streptococcus pneumoniae* and *Bacillus subtilis* group strains

**DOI:** 10.1186/s13568-017-0430-1

**Published:** 2017-06-20

**Authors:** Manabu Kitagawa, Tsukasa Shiraishi, Soh Yamamoto, Ryosuke Kutomi, Yasuo Ohkoshi, Toyotaka Sato, Hideki Wakui, Hideaki Itoh, Atsushi Miyamoto, Shin-ichi Yokota

**Affiliations:** 1 Department of Microbiology, Sapporo Medical University School of Medicine, Sapporo, 060-8556 Japan; 2 Department of Pharmaceutical Health Care and Sciences, Sapporo Medical University School of Medicine, Sapporo, 060-8543 Japan; 3 Department of Clinical Laboratory, NTT East Sapporo Hospital, Sapporo, 060-0061 Japan; 4 Department of Life Science, Graduate School and Faculty of Engineering Science, Akita University, Akita, 010-8502 Japan

**Keywords:** Natto, Antimicrobial peptide, *Streptococcus pneumoniae*, *Bacillus subtilis*, Subtilisin

## Abstract

We recently isolated a tumoricidal peptide from Natto, a Japanese traditional fermented food. In the present study, antimicrobial activity of the Natto peptide was examined. The peptide consisted of 45 amino acid residues, and its structure was predicted to be rich in α-helix. It excreted antimicrobial activity only against *Streptococcus pneumoniae* and *Bacillus subtilis* group (*B. subtilis*, *Bacillus pumilus*, and *Bacillus licheniformis*). Lesser antimicrobial activity was observed for *Streptococcus* species other than *S. pneumoniae*. Hemolysate or hemin was required for the antimicrobial activity of the peptide. The Natto peptide damages the cell membrane of *B. subtilis*. On the other hand, chain morphology was induced in *S. pneumoniae*, which is naturally diplococcus, during the early phases of the Natto peptide treatment; following that the cells were rapidly lysed. This suggested that the Natto peptide displayed a novel narrow spectrum of bactericidal activity and inhibited cell separation during cell division of *S. pneumoniae*.

## Introduction

Natto is a traditional Japanese food obtained after fermentation of boiled soybeans by *Bacillus subtilis*. It is recognized as a healthy food, and a rich source of vitamins, calcium, and various nutrients. Isoflavone, an analogue of estrogen, is derived from soybeans, and it is shown to prevent cerebral and myocardial stroke, breast cancer and prostate cancer (Zaheer and Humayoun Akhtar 2017). Lecithin is also derived from soybeans; it is a phospholipid containing unsaturated fatty acids. Lecithin has shown to prevent coronary artery diseases by preventing the deposition of cholesterol and other fats on the vascular endothelium and to prevent liver damages (Nicolosi et al. 2001). Nattokinase is a subtilisin family serine protease derived from *B. subtilis*. It exhibits a pronounced fibrinolytic activity, and is known as a blood thinner (Dabbagh et al. 2014; Sumi et al. 1990). Natto is the only food containing high levels of water-soluble vitamin K_2_ (menaquinone-7). The high vitamin K_2_ content of Natto results from fermentation; consequently, vitamin K_2_ content in Natto is over 100 times higher than in soybeans (Sakano et al. 1988). Natto, therefore, contains numerous useful substrates for human health derived from the soybeans, *B. subtilis,* and soybean fermentation products. We were interested in the new beneficial effects of the multifunctional food, Natto, on human health.

Recently, we discovered a new peptide (termed “Natto peptide”) that has cytotoxicity toward tumor cell lines but not toward normal cell lines (Hatakeyama et al. 2016). Injury of the cell membrane was suggested as a mechanism of this cytotoxicity. Such tumoricidal activity is also displayed by various antimicrobial peptides. One family of such antimicrobial peptides is the defensin family (Mattar et al. 2016; Suarez-Carmona et al. 2015). The defensin family peptides are disulfide-bond-rich cationic peptides found in both vertebrates and invertebrates. In human, α-defensins are mainly produced by neutrophils, and β-defensins are produced by leukocytes and epithelial cells. Another antimicrobial peptide is the cathelicidin family (Wang et al. 2014; Xhindoli et al. 2016). They are α-helix-rich peptides, with basic amino acids present every few residues. They are found in mammals, and are produced by neutrophils, macrophages, and keratinocytes. These cationic peptides display tumoricidal activity like as the Natto peptide and antimicrobial activity against various types of microorganisms, including Gram-positive bacteria, Gram-negative bacteria, and fungi (Mattar et al. 2016; Sang and Blecha 2008; Suarez-Carmona et al. 2015; Xhindoli et al. 2016). Given the above, we were interested in the antimicrobial activity of the Natto peptide.

## Materials and methods

### Preparation of the Natto peptide

Natto, which is commercially available, was donated by Yamada Foods (Misato, Akita Prefecture, Japan). Six Natto preparations were obtained, generated by using different *B. subtilis* strains. Natto was homogenized with Polytron PT4000 (Kinematica AG, Luzern, Switzerland) and centrifuged at 20,000×*g* for 15 min at 4 °C. Saturated ammonium sulfate was added to the supernatant at a concentration of 30%, and the mixture was stirred for 30 min at 4 °C and centrifuged at 20,000×*g* for 15 min at 4 °C. The supernatant was collected, and then saturated ammonium sulfate was added at a concentration of 50%. The mixture was stirred for 30 min at 4 °C and centrifuged at 20,000×*g* for 15 min at 4 °C. The resulting precipitate (30–50% saturated ammonium sulfate precipitated fraction) was dialyzed against 10 mM Tris–HCl (pH 7.4) for 24 h. The resulting material was precipitated with 25% saturated ammonium sulfate at 4 °C, and the supernatant was filtered through a 0.45 μm membrane filter. The filtrate was applied to a column of Butyl-Sepharose High Performance (GE Healthcare Life Sciences, Chalfont St Giles, UK) equilibrated with 10 mM Tris–HCl (pH 7.4) containing 25% saturated ammonium sulfate. The column was successively eluted with 25, 20, 15, 10, 5 and 2% saturated ammonium sulfate in 10 mM Tris–HCl (pH 7.4). The eluent with 10% saturated ammonium sulfate was dialyzed in 10 mM Tris–HCl (pH 7.4), lyophilized, and used as a Natto peptide preparation. The Natto peptide was yielded approximately 1 g from 500 g of Natto.

The purity of the peptide was confirmed by Tris/Tricine-sodium dodecyl sulfate–polyacrylamide gel electrophoresis (Tris/Tricine-SDS-PAGE) (Schägger and von Jagow 1987) and Coomassie Brilliant Blue (CBB) staining.

The protein concentration was determined by the method of Bradford (1976) using bovine serum albumin as a standard.

### Amino acid sequence of the Natto peptide

For amino acid sequence determination, the Natto peptide preparation was further purified by column chromatography on Wakosil-5C18HG column (Wako Pure Chemical, Tokyo, Japan). The peptide was eluted with acetonitrile [linear gradient of 0–60% (v/v)]. The fraction eluted with approximately 30% acetonitrile was pooled and lyophilized. The resulting material was analyzed. Amino acid sequence of the peptide was determined using the protein sequencer PPSQ-31B/33B (Shimadzu, Kyoto, Japan).

### Bacterial strains

*Bacillus subtilis*, AHU 1035, AHU 1037, AHU 1604, AHU 1615, AHU 1708^T^ and AHU 1722, *Bacillus cereus* AHU 1358, *Bacillus licheniformis* AHU 1371, *Bacillus megaterium* AHU 1373, and *Bacillus pumilus* AHU 1386 were obtained from the Research Faculty of Agriculture, Hokkaido University (Sapporo, Japan). *B. cereus* JCM 2152^T^, *Streptococcus agalactiae* JCM 5671^T^, *Streptococcus mitis* JCM 12971^T^, *Streptococcus mutans* JCM 5705^T^, *Streptococcus pyogenes* JCM 5674^T^, *Streptococcus salivarius* JCM 5707^T^, and *Streptococcus sanguinis* JCM 5708^T^ were obtained from the Japan Collection of Microorganisms, Riken BioResource Center (Tsukuba, Japan). *Acinetobacter baumannii* ATCC 19606^T^, *Escherichia coli* ATCC 25922 and ATCC 35218, *Lactobacillus fermentum* ATCC 9338, *Lactobacillus rhamnosus* ATCC 7469^T^, *Pseudomonas aeruginosa* ATCC 27853, *Staphylococcus aureus* ATCC 25923, *Streptococcus pneumoniae* ATCC 49619, and *Haemophilus influenzae* ATCC 51907 were obtained from the American Type Culture Collection (Manassas, VA, USA). *Lactobacillus plantarum* NRIC 1067^T^ was obtained from the NODAI Culture Collection Center, Tokyo University of Agriculture (Tokyo, Japan). *A. baumannii* SRAC2, *S. aureus* SUNA1 [hospital-acquired methicillin-resistant *S. aureus* (MRSA)] and SR-581 (community-acquired MRSA), *Enterococcus faecium* M2483, *Enterococcus faecalis* HU1 [vancomycin-resistant *Enterococcus* (VRE)] and M2486, *E. coli* 7249 (ST131), *P, aeruginosa* PA103 and 9728 (mucoid), *Serattia marcescens* SRSM2, *S. pneumoniae* SR11, MH101 [penicillin-resistant *S. pneumoniae* (PRSP)], 237 (PRSP) and 442 (mucoid), and *Candida albicans* SRCA1 and SRCA4 were clinical isolates stocked in our laboratory. *S. pneumoniae* R6 and P103 [*lytA*-deficient mutant derived from R6 (Δ*lytA*)] were kindly provided by Dr. Ernesto García (Centro de Investigaciones Biológicas, CSIC, Madrid, Spain) (Moscoso et al. 2014).

### Minimum inhibitory concentration

Antimicrobial activity was estimated by minimum inhibitory concentration (MIC) determinations using a broth microdilution procedure basically according to a guideline of the Clinical Laboratory Standards Institute (14). Briefly, cell suspensions (5 × 10^5^ cells/mL) and serially diluted solutions of the Natto peptide were mixed in a 96 well microplate, and cultured at 37 °C for 24 h. The basal medium was Mueller-Hinton (MH) broth (Becton–Dickinson, Franklin Lakes, NJ, USA) or Todd Hewitt (TH) broth (Becton–Dickinson) supplemented with 5% (v/v) horse hemolysate (Nippon Bio-Test Laboratories, Tokyo, Japan).

### The combined effect of the Natto peptide and other antibacterial agents

Cefditoren and tebipenem were provided by Meiji Seika Pharma (Tokyo, Japan). Clarithromycin was provided by Taisho Pharmaceutical (Tokyo, Japan). Amoxicillin, amikacin, tobramycin, and gentamicin were purchased from Wako Pure Chemical. Levofloxacin was purchased from LKT Laboratories (St. Paul, MN, USA). A combined effect was defined according to the fractional inhibitory concentration (FIC) index deduced from MIC values, as follows: synergistic effect, FIC ≤ 0.5; additive effect, FIC 0.6–1.0; no effect, FIC 1.1–2; and antagonistic effect, FIC > 2.

### The effect of supplements on the antimicrobial activity of the Natto peptide

Hemin was purchased from Wako Pure Chemical. Horse serum was from Thermo Fisher Scientific (Waltham, MA, USA). Heat-treated horse hemolysate was prepared by heating at 100 °C for 10 min; various concentrations were suspended in TH broth, and then centrifuged at 9000×*g* for 5 min. The supernatant was filtered (0.22 µm) and used in culture media for MIC measurements. The effect on antimicrobial activity was determined by MIC measurements using the broth microdilution method. Briefly, suspensions of *S. pneumoniae* R6 cells (5 × 10^5^ cells/mL) in TH broth containing serial dilutions of the Natto peptide and serial dilutions of the supplements were cultured at 37 °C for 24 h.

### The effect of the Natto peptide on bacterial cell growth and survival

*Streptococcus pneumoniae* and *B. subtilis* cells were precultured on TH agar containing 5% (v/v) horse hemolysate at 37 °C for 12 h. The cells were then suspended in saline, and used to inoculate TH broth containing 5% (v/v) horse hemolysate at an optical density at 625 nm of 0.000625 (approximately 1 × 10^5^ cells/mL). The Natto peptide was added to the cell cultures at the time of inoculation or 4 h after the inoculation (approximately 1 × 10^7^ cells/mL). Cell numbers were determined by plating aliquots of the cultures sampled at various times on TH agar containing 5% (v/v) horse hemolysate, and culturing under microaerophilic conditions; the resulting colonies were then counted.

### Morphological observations of the Natto peptide treated cells

*Streptococcus pneumoniae* and *B. subtilis* cells (control cells or cells treated with the Natto peptide) were observed by transmission electron microscopy (TEM) after negative staining as previously described (O’Connell Motherway et al. 2011), with minor modifications. The bacterial cells were cultured in TH broth containing 5% (v/v) horse hemolysate at 37 °C for 4 or 8 h in the presence or absence of the Natto peptide. Cells were fixed with 1% (v/v) glutaraldehyde for 30 min at 4 °C. Copper grids (150 mesh; Veco B.V., Eerbeek, the Netherlands) were floated on the fixed cell suspensions for 10 min at room temperature. The grids were washed once for 5 min in 0.02 M glycine-phosphate-buffered saline, and three times for 5 min in ultra-pure water. Negative staining was performed using an EM stainer (Nisshin EM, Tokyo, Japan). The grids were observed using JEOL JEM-1400 TEM (JEOL, Tokyo, Japan) operated at 80 kV.

## Results

### Characterization of the Natto peptide

**Fig. 1:**
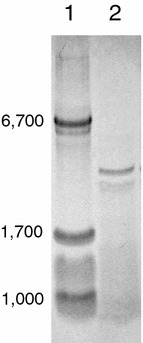
Tirs/Tricine-SDS-PAGE analysis of the Natto peptide preparation. The sample was applied on a 15–20% gradient polyacrylamide gel. *Lane 1* molecular weight standards. *Lane 2* Natto peptide preparation at 10 μg/lane

**Fig. 2:**
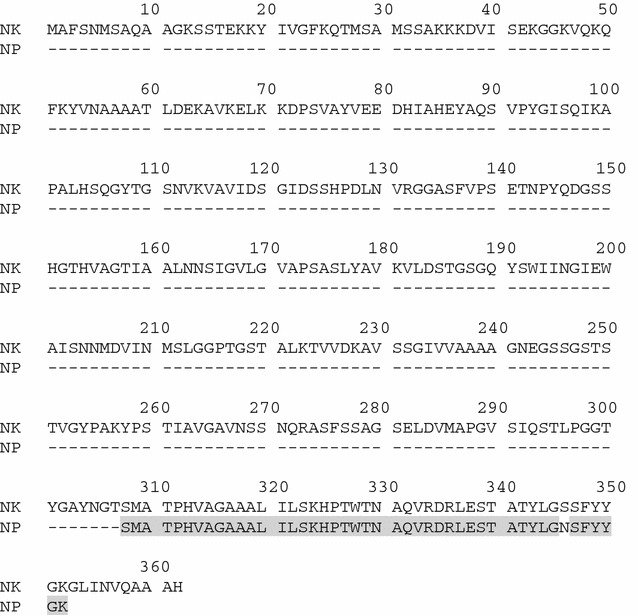
Comparison of amino acid sequence of nattokinase (NK) and Natto peptide (NP). The amino acid sequence of NK is cited from ACJ48969.1

The Natto peptide was successfully isolated from the active fraction of homogenate of Natto by successive ammonium sulfate precipitation steps and hydrophobic interaction chromatography (Hatakeyama et al. 2016). The resulting peptide migrated as a single band of approximately 5 kDa, as estimated by Tris/Tricine-SDS-PAGE and CBB staining (Fig. 1). The Natto peptide consisted of 45 amino acid residues and its sequence was shown in Fig. 2. It shared extremely high homology with a sequence near the C-terminal side of subtilisin family proteins, which are serine proteases produced by *Bacillus* species. High, 97.8%, homology with nattokinase of *B. subtilis* (GenBank ACJ48969.1) (Fig. 2) and 80.0% homology with keratinase of *B. licheniformi*s (GenBank AID16241.1) were observed.

### Antimicrobial activity of the Natto peptide

The antimicrobial activity of the Natto peptide was determined by MIC measurements with various microorganisms. The MH medium was first used in the assays, according to the CLSI recommendations. The Natto peptide showed antimicrobial activity only *S. pneumoniae* (data not shown). MIC for *S. pneumoniae* was determined using MH broth supplemented with 5% (v/v) hemolysate (14). Next, MIC values were determined using TH broth, a medium more rich than MH broth, supplemented with 5% (v/v) horse hemolysate (Table 1). The Natto peptide displayed antimicrobial activity against *S. pneumoniae*, including PRSP and mucoid strains, and *B. subtilis*, *B. pumilus*, and *B. licheniformis*, at a concentration of 8 or 16 μg/mL. Lesser activity was observed against strains of *Streptococcu*s species other than *S. pneumoniae* and against *L. fermentum* at a concentration of 64 or 128 μg/mL. The Natto peptide did not display any antimicrobial activity against other Gram-positive bacteria, including *B. cereus* and *B. megaterium*, any of the Gram-negative bacteria examined, and *C. albicans*.

**Table 1:** MICs of the Natto-peptide tested against various Gram-positive bacteria, Gram-negative bacteria, and fungi

Strain	MIC (μg/mL)
Gram-positive bacteria	
*B. cereus* JCM 2152^T^	>128
*B. cereus* AHU 1358	>128
*B. licheniformis* AHU 1371	8
*B. megaterium* AHU 1373	>128
*B. pumilus* AHU 1386	8
*B. subtilis* AHU 1708^T^	8
*B. subtilis* AHU 1035	16
*B. subtilis* AHU 1037	8
*B. subtilis* AHU 1604	8
*B. subtilis* AHU 1615	16
*B. subtilis* AHU 1722	16
*E. faecalis* HU1 (VRE)	>128
*E. faecalis* M2486	>128
*E. faecium* M2483	>128
*L. fermentum* ATCC 9338	64
*L. plantarum* NRIC 1067^T^	>128
*L. rhamnosus* ATCC 7469^T^	>128
*S. aureus* ATCC 25923	>128
*S. aureus* SUNA1 (HA-MRSA)	>128
*S. aureus* SR-581(CA-MRSA)	>128
*S. agalactiae* JCM 5671^T^	64
*S. mitis* JCM 12971^T^	64
*S. mutans* JCM 5705^T^	64
*S. pneumoniae* ATCC 49619	8
*S. pneumoniae* R6	8
*S. pneumoniae* P103 (Δ*lytA*)	8
*S. pneumoniae* SR11	16
*S. pneumoniae* MH101 (PRSP)	16
*S. pneumoniae* 237 (PRSP)	8
*S. pneumoniae* 442 (mucoid)	8
*S. pyogenes* JCM 5674^T^	128
*S. salivarius* JCM 5707^T^	128
*S. sanguinis* JCM 5708^T^	64
Gram-negative bacteria	
*A. baumannii* ATCC 19606^T^	>128
*A. baumannii* SRAC2	>128
*E. coli* ATCC 25922	>128
*E. coli* ATCC 35218	>128
*E. coli* 7249 (ST131)	>128
*H. influenza* ATCC 51907	>128
*P. aeruginosa* ATCC 27853	>128
*P. aeruginosa* PA103	>128
*P. aeruginosa* 9728 (mucoid)	>128
*S. marcescens* SRSM2	>128
Fungi	
*C. albicans* SRCA1	>128
*C. albicans* SRCA4	>128

Protein concentrations were adjusted by the results of colorimetric assay as described in “Materials and methods”

Natto peptides obtained from various Natto preparations that were prepared using different soybeans and different *B. subtilis* strains also displayed specific antimicrobial activity against *S. pneumoniae* and *B. subtilis*, however, the activities were different in some instances (Table 2). This indicated that the Natto peptide was typically and commonly present in Natto.

**Table 2:** Antimicrobial activity of the Natto peptide from various Natto preparations obtained using different soybeans and *B. subtilis* strains

Natto preparation lot No.	*B. subtilis* strain used for starter	MIC (μg/mL)	
		*S. pneumoniae* R6	*B. subtilis* AHU 1708^T^
160	S63	64	128
170	H23	8	16
180	S125	8	16
200	S278	8	8
300	S125-5	32	32

### The combined effect of the Natto peptide and other antimicrobial agents

Next, the combined effect of the Natto peptide and antimicrobial agents was examined. The tested antimicrobials were aminoglycosides (that injure the cell membrane in addition to inhibition of protein synthesis) and drugs frequently used in pneumococcal infections. The antimicrobial agents examined did not affect (synergistically, additively or antagonistically) to the Natto prptide activity, as assessed by the FIC index analysis (Table 3).

**Table 3:** Combined antimicrobial effect (FIC index) of the Natto peptide and other antimicrobial agents

Antibiotic	FIC index
Amoxicillin	2
Cefditoren	2
Tebipenem	2
Levofloxacin	2
Clarithromycin	1.38
Amikacin	1.09
Tobramycin	2
Gentamicin	2

### Hemin is required for antimicrobial activity of the the Natto peptide

Horse hemolysate was required for the occurrence of antimicrobial activity of the Natto peptide in a dose-dependent manner (Table 4). Heat treatment (100 °C, 10 min) of horse hemolysate resulted in the aggregation of many proteins present in the hemolysate. The aggregates were removed by centrifugation and filtration. The resulting supernatant retained the ability to support the antimicrobial activity of the Natto peptide. This suggested that heat-stable substance(s) present in the horse hemolysate were required for the antimicrobial activity of the peptide. Horse serum did not alter the antimicrobial activity of the peptide, therefore, blood cell component(s) were subsequently evaluated. Hemin is used as a supplement in MIC measurements with *H. influenzae* as the X factor (White and Granick 1963). The antimicrobial activity of the Natto peptide against *S. pneumoniae* was supported by hemin in a concentration-dependent manner (Table 4). The amount of hemin required for the occurrence of the antimicrobial activity was comparable with the amount of heme (5–6 mg/L) in horse hemolysate required for the occurrence of the antimicrobial activity.

**Table 4:** The effect of medium supplemented with horse hemolysate, hose serum, and hemin on the MIC of the Natto peptide with *S. pneumoniae* R6 strain

Supplement	Concentration^b^	MIC (μg/mL)
None	–	>128
Horse hemolysate^a^	5%	8
	1%	4
	0.32%	4
	0.10%	8
	0.032%	32
	0.010%	64
	0.0032%	128
	0.0010%	>128
Supernatant of heat-treated horse hemolysate	20%	4
	5%	32
Horse serum	5%	>128
Hemin^a^	6.9 μg/mL	8
	3.4 μg/mL	32
	1.7 μg/mL	64
	0.86 μg/mL	128
	0.43 μg/mL	>128

^a^Hose hemolysate [5%(v/v)] and hemin (6.9 μg/mL) did not any antimicrobial effects against *S. pneumoniae*

^b^The percent concentrations are (v/v)

### The effect of the Natto peptide on bacterial cell growth and survival

**Fig. 3:**
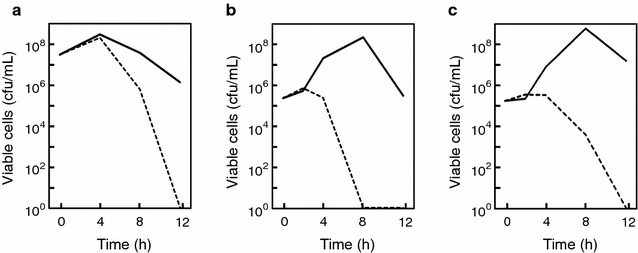
Survival curves of *S. pneumoniae* strains in the presence and absence of the Natto peptide. **a** *S. pneumoniae* R6 cells were pre-cultured to a cell density of approximately 10^7^ cells/mL in TH broth containing 5% (v/v) horse hemolysate. The Natto peptide was then added at a concentration of 64 μg/mL. **b**, **c** *S. pneumoniae* R6 (**b**) and P103 (*lytA*-deficient mutant derived from R6) (**c**) cells were suspended at a density of approximately 10^5^ cells/mL in TH broth containing 5% (v/v) horse hemolysate. The Natto peptide was then added at a concentration of 64 μg/mL. Throughout the cultivation, the cultures were sampled, and culture aliquots were spread on a TH agar supplemented with 5% (v/v) horse hemolysate, and cultured for 24 h. Grown colonies were then counted. *Dashed lines* indicate cell numbers in the presence of the Natto peptide. *Solid lines* indicate cell numbers in the absence of the Natto peptide. Each experiment performed at least three times, and the representative results are presented. The similar results were observed in the same experiments

Survival curves of *S. pneumoniae* and *B. subtilis* cell cultures in the presence or absence of the Natto peptide were examined. The growth of *S. pneumoniae* was similar during the early phases of growth in the presence and absence of the Natto peptide (Fig. 3). When high cell numbers were used in the inoculum (approximately 10^7^ cells/mL), the cultures reached maximum cell density (approximately 10^8^ cells/mL) regardless of the presence of the Natto peptide; the number of viable cells then decreased (Fig. 3a). The rate of the decrease of viable cell numbers was markedly higher in the presence of the Natto peptide than in the absence of the peptide. When low cell numbers were used in the inoculum (approximately 10^5^ cells/mL), in the absence of the peptide, the culture reached maximum cell density (approximately 10^8^ cells/mL) after 8 h, following which the viable cell numbers decreased. On the other hand, in the presence of the peptide, the culture did not reach maximum cell numbers and the number of viable cells rapidly decreased 2 h after the addition of Natto peptide (Fig. 3b). These results suggested that *S. pneumoniae* cells initially grew normally in the presence of the Natto peptide; the growth was then terminated and the cells rapidly lysed. The rapid decrease of viable *S. pneumoniae* cells seemed to be associated with autolysis. Consequently, the contribution of LytA, the main *S. pneumoniae*-specific autolysin, to the decrease of viable cells in late phase was examined, using the *lytA*-deficient mutant P103. The rate of viable cell number decrease in the presence of the Natto peptide was lower in P103 than in the parent strain R6 (Fig. 3b, c). This indicated that the autolysis caused by LytA enhanced the bactericidal rate, but it was not essential for the antimicrobial activity of the Natto peptide.

**Fig. 4:**
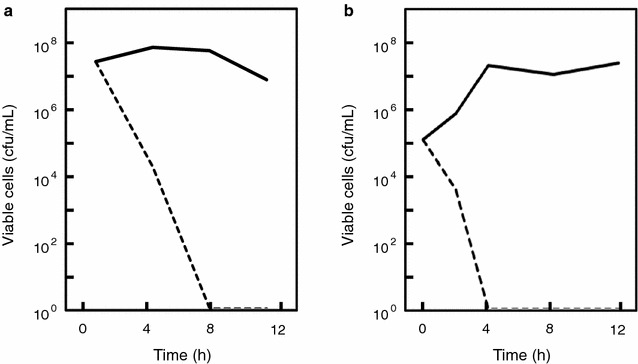
Survival curves of *B. subtilis* strain in the presence and absence of the Natto peptide. **a** *B. subtilis* AHU 1708^T^ cells were pre-cultured to a cell density of approximately 10^7^ cells/mL in TH broth containing 5% (v/v) horse hemolysate. The Natto peptide was then added at a concentration of 64 μg/mL. **b** *B. subtilis* AHU 1708^T^ cells were suspended at a cell density of approximately 10^5^ cells/mL in TH broth containing 5% (v/v) horse hemolysate. The Natto peptide was then added at a concentration of 64 μg/mL, and the cells were cultured. Throughout the cultivation, the cultures were sampled, and culture aliquots were spread on TH agar supplemented with 5% (v/v) horse hemolysate, and cultured for 24 h. Grown colonies were then counted. *Dashed lines* indicate cell numbers in the presence of the Natto peptide. *Solid lines* indicate cell numbers in the absence of the Natto peptide. Each experiment performed at least three times, and the representative results are presented. The similar results were observed in the same experiments

The survival curves of *B. subtilis* cells also indicated that viable cell numbers rapidly decreased in the presence of the Natto peptide (Fig. 4). The decrease occurred immediately after the addition of the Natto peptide, and seen when both high (approximately 10^7^ cells/mL; Fig. 4a) and low (approximately 10^5^ cells/mL; Fig. 4b) inocula were used. This suggested that the antibacterial action of the Natto peptide differed in *S. pneumoniae* and *B. subtilis*.

### Morphological observations of cells treated with the Natto peptide

**Fig. 5:**
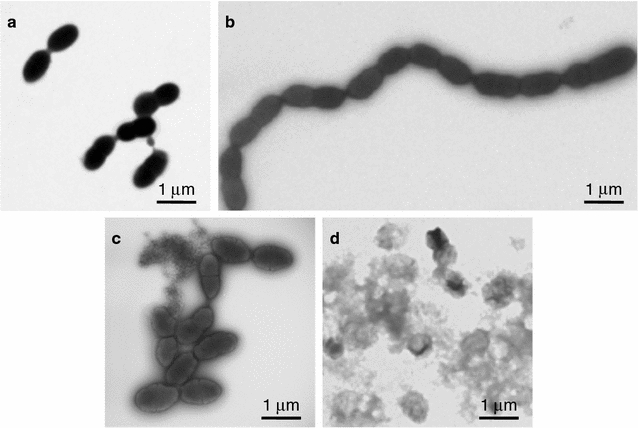
TEM analysis with negative staining of *S. pneumoniae* R6 cells cultured in the presence or absence of the Natto peptide. The cells were cultured in TH broth containing 5% (v/v) horse hemolysate in the absence (**a**, **c**) or presence (**b**, **d**) of the Natto peptide (64 μg/mL). After 4 h (**a**, **b**) or 8 h (**c**, **d**) of incubation, the cells were harvested and observed TEM after negative staining

*Streptococcus pneumoniae* cell morphology was examined by TEM after negative staining (Fig. 5). In the absence of the Natto peptide, *S. pneumoniae* cells looked like typical diplococci (Fig. 5a). The cells formed chain-like structure 4 h after the addition of the Natto peptide (Fig. 5b). These results suggested that the cells failed to separate after cell division in the presence of the peptide. After 8 h of culture, the observed cell lysis was more pronounced in the presence than the absence of the Natto peptide (Fig. 5c, d).

**Fig. 6:**
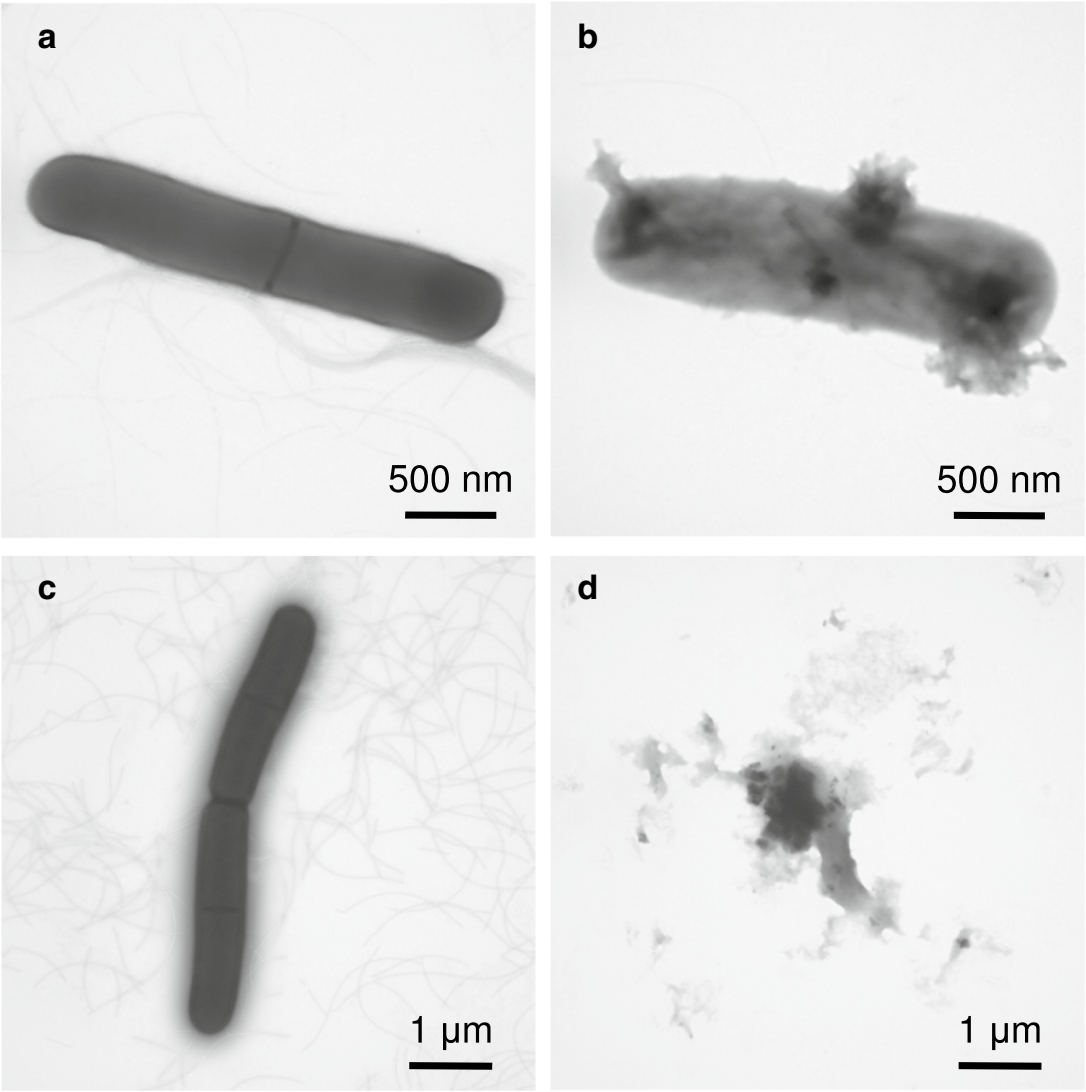
TEM analysis with negative staining of *B. subtilis* AHU 1708^T^ cells cultured in the presence or absence of the Natto peptide. The cells were cultured in TH broth containing 5% (v/v) hemolysate in the absence (**a**, **c**) or presence (**b**, **d**) of the Natto peptide (64 μg/mL). After 4 h (**a**, **b**) or 8 h (**c**, **d**) of incubation, the cells were harvested and observed using TEM after negative staining

In the case of *B. subtilis* (Fig. 6), localized cell membrane damages and cell swelling were observed 4 h after that Natto peptide addition (Fig. 6b). After 8 h when no viable cells were seen, the cells completely lysed (Fig. 6d).

## Discussion

Recently, we reported a tumoricidal activity of the Natto extract; approximately 5 kDa peptide was identified as the active component (Hatakeyama et al. 2016). As shown here, the peptide consisted of 45 amino acid residues, and shared extremely high homology with the C-terminal region of a subtilisin family serine protease, Nattokinase produced by *B. subtilis*. The structure of the peptide is probably rich in α-helix deduced from secondary structure predictions and the presence of basic amino acid residues appearing every few residues. Such structural characteristics are found in several types of antimicrobial peptides (Yount et al. 2006). Typically, the characters were found in cathelicidin family antimicrobial peptides (Wang et al. 2014; Xhindoli et al. 2016). The cathelicidin antimicrobial peptides generally have broad spectrum of antimicrobial activity and are effective against multidrug-resistant bacteria (Sang and Blecha 2008; Zanetti et al. 2002). They also show cytotoxicity toward tumor cell lines, but not toward normal cell lines, similarly to the Natto peptide. Thus, we anticipated that the Natto peptide possesses a broad-spectrum antimicrobial activity with broad spectrum, similarly to the antimicrobial peptides.

Unexpectedly, we found a novel antimicrobial activity of the Natto peptide. The spectrum of activity was very narrow, namely, only against *S. pneumoniae* and several *Bacillus* strains (Table 1). Little antimicrobial activity was observed against *Streptococcus* spp., including group A (*S. pyogenes*) and group B (*S. agalactiae*) streptococci and oral viridans streptococci. The Natto peptide seemed to potentially possess an antimicrobial activity against streptococci, and most pronouncedly against *S. pneumoniae*. On the other hand, the Natto peptide showed antimicrobial activity against *B. subtilis*, *B. licheniformis*, and *B. pumilus*, no antimicrobial activity was observed for *B. cereus* and *B. megaterium*. *B. subtilis*, *B. pumilus*, and *B. licheniformis* are phylogenetically classified into the *B. subtilis* group (Bhandari et al. 2013; Turenne et al. 2015); *B. cereus* and *B. megaterium* are phylogenetically distant from the *B. subtilis* group. The results indicated that the antimicrobial activity of the Natto peptide was specific for *S. pneumoniae* and the *B. subtilis* group of the genus *Bacillus*.

The antimicrobial activity was commonly observed various Natto preparations produced by different strains of *B. subtilis* (Table 2), whereas levels of the activity was different each other. This could be due to differences of expression levels and/or amino acid sequences of the peptides; however, this is a future issue.

The specific antimicrobial action point(s) of the Natto peptide is very interesting, i.e., the early growth phase after the addition of the peptide. Comparable *S. pneumoniae* cell growth was observed at early time points, regardless of whether the peptide was added. The *S. pneumoniae* cells formed long chains in the presence of the Natto peptide, although *S. pneumoniae* is naturally diplococcus as seen in the absence of the peptide (Fig. 5). The results suggested that the Natto peptide caused a failure of cell separation after cell division. At late growth phases, viable cell numbers rapidly decreased in the presence of the peptide (Fig. 3). We proposed that the cells were dead by autolysis. The rapid and pronounced cell death was attributable to LytA, which is a major autolysin specific for *S. pneumoniae* (Maestro and Sanz 2016); however, LytA was not a direct target of the anti-*S. pneumoniae* bactericidal activity of the Natto peptide. Similar long chain morphology of *S. pneumoniae* is found in cells treated with choline (Maestro et al. 2007). Choline interacts with several pneumococcal proteins and affects autolysis and cell division/separation (Maestro and Sanz 2016). Chain morphology is also observed in a defective mutant of LytB, a peptidoglycan hydrolase contributing to cell separation (Bai et al. 2014; De Las Rivas et al. 2002).

The current results suggest that the Natto peptide might be a fragment of subtilisin family protein, namely Nattokinase. It might be generated by a proteolytic cleavage; however, the responsible protease was not identified. The bactericidal activity against *B. subtilis* was immediately apparent following the addition of the Natto peptide, and the cells died because of a direct membrane injury (Fig. 6). This means that the production of the Natto peptide constitutes a suicide mechanism of *B. subtilis* group bacteria. Its mode of action has not been characterized and should be the focus of future research.

Hemolysate or hemin was required for the antimicrobial activity of the Natto peptide against both *S. pneumoniae* and *B. subtilis* group strains (Table 4). Heme/hemin could alter the secondary structure of the peptide; however, further examination is necessary to verify this hypothesis.

In conclusion, we identified a unique antimicrobial peptide with a narrow spectrum of activity in Natto extract. *S. pneumoniae* has great impact in the clinic. It causes acute otitis media and sinusitis mainly in children, community-acquired pneumonia mainly in elders, and meningitis as an invasive infection (Hamborsky et al. 2015). Vaccines against *S. pneumoniae* are used for the prevention of invasive infection in children and community-acquired pneumonia in elders. Furthermore, antimicrobial resistant *S. pneumoniae*, such as PRSP, are increasing, and their treatment is now problematic. *S. pneumoniae*-specific antimicrobials would be promising therapeutic agents, as such agents would not disturb the normal flora; however, peptides are usually difficult to employ as therapeutic agents because of their potentially low stability and bioavailability. Thus the molecular mechanisms of the anti-pneumococcal activity of the Natto peptide should be evaluated for application in therapeutic agents for *S. pneumoniae* infection.

## Abbreviations

CBB: Coomassie Brilliant Blue
FIC: fractional inhibitory concentration
MH: Mueller–Hinton
MIC: minimum inhibitory concentration
MRSA: methicillin-resistant *Staphylococcus aureus*
PRSP: penicillin-resistant *Streptococcus pneumoniae*
SDS-PAGE: sodium dodesyl sulfate–polyacrylamide gel electrophoresis
TEM: transmission electron microscopy
TH: Todd-Hewitt
VRE: vancomycin-resistant *Enterococcus*

## References

[CR1] Bai XH, Chen HJ, Jiang YL, Wen Z, Huang Y, Cheng W, Li Q, Qi L, Zhang JR, Chen Y, Zhou CZ (2014). Structure of pneumococcal peptidoglycan hydrolase LytB reveals insights into the bacterial cell wall remodeling and pathogenesis. J Biol Chem.

[CR2] Bhandari V, Ahmod NZ, Shah HN, Gupta RS (2013). Molecular signatures for *Bacillus* species: demarcation of the *Bacillus subtilis* and *Bacillus cereus* clades in molecular terms and proposal to limit the placement of new species into the genus *Bacillus*. Int J Syst Evol Microbiol.

[CR3] Bradford MM (1976). A rapid and sensitive method for the quantitation of microgram quantities of protein utilizing the principle of protein-dye binding. Anal Biochem.

[CR4] Dabbagh F, Negahdaripour M, Berenjian A, Behfar A, Mohammadi F, Zamani M, Irajie C, Ghasemi Y (2014). Nattokinase: production and application. Appl Microbiol Biotechnol.

[CR5] De Las Rivas B, García JL, López R, García P (2002). Purification and polar localization of pneumococcal LytB, a putative endo-β-*N*-acetylglucosaminidase: the chain-dispersing murein hydrolase. J Bacteriol.

[CR6] Hamborsky J, Kroger A, Wolfe S, Hamborsky J, Kroger A, Wolfe S (2015). Pneumococcal disease. Centers for Disease Control and Prevention. Epidemiology and prevention of vaccine-preventable diseases.

[CR7] Hatakeyama S, Kafuku M, Okamoto T, Kakizaki A, Shimasaki N, Fujie N, Takahashi S, Nakayama M, Tagawa H, Komatsuda A, Grave E, Wakui H, Itoh H (2016). Studies on the anticancer mechanisms of the Natto extract. J Soc Mater Eng Resour Jpn.

[CR8] Maestro B, Sanz JM (2016). Choline binding proteins from *Streptococcus pneumoniae*: a dual role as enzybiotics and targets for the design of new antimicrobials. Antibiotics.

[CR9] Maestro B, González A, García P, Sanz JM (2007). Inhibition of pneumococcal choline-binding proteins and cell growth by esters of bicyclic amines. FEBS J.

[CR10] Mattar EH, Almehdar HA, Yacoub HA, Uversky VN, Redwan EM (2016). Antimicrobial potentials and structural disorder of human and animal defensins. Cytokine Growth Factor Rev.

[CR11] Moscoso M, Esteban-Torres M, Menéndez M, García E (2014). *In vitro* bactericidal and bacteriolytic activity of ceragenin CSA-13 against planktonic cultures and biofilms of *Streptococcus pneumoniae* and other pathogenic streptococci. PLoS ONE.

[CR12] Nicolosi RJ, Wilson TA, Lawton C, Handelman GJ (2001). Dietary effects on cardiovascular disease risk factors: beyond saturated fatty acids and cholesterol. J Am Coll Nutr.

[CR13] O’Connell Motherway M, Zomer A, Leahy SC, Reunanen J, Bottacini F, Claesson MJ, O’Brien F, Flynn K, Casey PG, Munoz JA, Kearney B, Houston AM, O’Mahony C, Higgins DG, Shanahan F, Palva A, de Vos WM, Fitzgerald GF, Ventura M, O’Toole PW, van Sinderen D (2011). Functional genome analysis of *Bifidobacterium breve* UCC2003 reveals type IVb tight adherence (Tad) pili as an essential and conserved host-colonization factor. Proc Natl Acad Sci USA.

[CR14] Sakano T, Notsumoto S, Nagaoka T, Morimoto A, Fujimoto K, Masuda S, Suzuki Y, Hirauchi K (1988). Measurement of K vitamins in food by high-performance liquid chromatography with fluorometric detection. Vitamins.

[CR15] Sang Y, Blecha F (2008). Antimicrobial peptides and bacteriocins: alternatives to traditional antibiotics. Anim Health Res Rev.

[CR16] Schägger H, von Jagow G (1987). Tricine-sodium dodecyl sulfate-polyacrylamide gel electrophoresis for the separation of proteins in the range from 1 to 100 kDa. Anal Biochem.

[CR17] Suarez-Carmona M, Hubert P, Delvenne P, Herfs M (2015). Defensins: “Simple” antimicrobial peptides or broad-spectrum molecules?. Cytokine Growth Factor Rev.

[CR18] Sumi H, Hamada H, Nakanishi K, Hiratani H (1990). Enhancement of the fibrinolytic activity in plasma by oral administration of nattokinase. Acta Haematol.

[CR19] Turenne CT, Snyder JW, Alexander DC, Carroll KC, Funke G, Landry ML, Richter SS, Warnock DW (2015). *Bacillus* and other aerobic endspore-forming bacteria. Manual of clinical microbiology.

[CR20] Wang G, Mishra B, Epand RF, Epand RM (2014). High-quality 3D structures shine light on antibacterial, anti-biofilm and antiviral activities of human cathelicidin LL-37 and its fragments. Biochim Biophys Acta.

[CR21] White DC, Granick S (1963). Hemin biosynthesis in *Haemophilus*. J Bacteriol.

[CR22] Xhindoli D, Pacor S, Benincasa M, Scocchi M, Gennaro R, Tossi A (2016). The human cathelicidin LL-37-A pore-forming antibacterial peptide and host-cell modulator. Biochim Biophys Acta.

[CR23] Yount NY, Bayer AS, Xiong YQ, Yeaman MR (2006). Advances in antimicrobial peptide immunobiology. Biopolymers.

[CR24] Zaheer K, Humayoun Akhtar M (2017). An updated review of dietary isoflavones: nutrition, processing, bioavailability and impacts on human health. Crit Rev Food Sci Nutr.

[CR25] Zanetti M, Gennaro R, Skerlavaj B, Tomasinsig L, Circo R (2002). Cathelicidin peptides as candidates for a novel class of antimicrobials. Curr Pharm Des.

